# Determination of Blood NOTCH3 Extracellular Domain and Jagged-1 Levels in Healthy Subjects

**DOI:** 10.3390/ijms231810547

**Published:** 2022-09-11

**Authors:** Hyesung Kim, Bogun Jang, Yang-Ji Kim, Jay Chol Choi

**Affiliations:** 1 Department of Pathology, School of Medicine, Jeju National University, Jeju 63241, Korea; 2 Department of Pathology, Jeju National University Hospital, Jeju 62341, Korea; 3 Institute for Medical Science, Jeju National University, Jeju 63241, Korea; 4 Department of Neulogy, Jeju National University Hospital, Jeju 62341, Korea; 5 Department of Neurology, School of Medicine, Jeju National University, Jeju 63241, Korea

**Keywords:** NOTCH3 extracellular domain, Jagged-1, CADASIL, blood marker, serum, plasma

## Abstract

Cerebral autosomal dominant arteriopathy with subcortical infarcts and leukoencephalopathy (CADASIL) is the most common genetic disorder among those responsible for hereditary strokes, and it is caused by a mutation in the *NOTCH3* gene on chromosome 19. Blood biomarkers related to the Notch signaling pathway have not been investigated extensively in CADASIL. In this study, we measured the serum and plasma levels of NOTCH3 extracellular domain (N3ECD) and its ligand, Jagged-1, in 279 healthy subjects. The levels of N3ECD and Jagged-1 showed significant correlations in both serum (*p* < 0.0001, *r* = 0.2681) and plasma (*p* < 0.0001, *r* = 0.4065). The N3ECD levels were significantly higher in the serum than in plasma and tend to increase with age. In contrast, there was no significant difference between the serum and plasma levels of Jagged-1 levels. To summarize, we were able to measure N3ECD and Jagged-1 protein levels in healthy human serum and plasma. Taken together, our findings provide the basis for further studies investigating the clinical use of blood N3ECD and Jagged-1 levels for CADASIL and other Notch signaling-related diseases.

## 1. Introduction

Notch signaling pathways are evolutionarily conserved intercellular signaling mechanisms involved in vascular development and physiology in vertebrates [1]. There are four Notch receptors and five ligands (Jagged-1, Jagged-2, Delta-like 1, Delta-like 3, Delta-like 4) in humans, and the expression of the receptors and ligands varies among different cell types. In vascular smooth muscle cells (VSMC), the Notch signaling plays a crucial role in the cell differentiation and the response to vascular injury [2,3]. Mutations in the *NOTCH3* gene located on the chromosome 19 cause a hereditary stroke disorder known as cerebral autosomal dominant arteriopathy with subcortical infarcts and leukoencephalopathy (CADASIL) [4]. CADASIL is the most common hereditary stroke disorder and mainly affects VSMC in the small blood vessels throughout the body [5,6,7]. In humans, the *NOTCH3* gene consists of 33 exons and encodes a single-pass transmembrane receptor of 2321 amino acids. The NOTCH3 receptor consists of a large extracellular domain (ECD) with 34 tandem epidermal growth factor (EGF)-like repeats, three Notch/Lin12 repeats, a transmembrane domain, and an intracellular domain [4]. Under normal conditions, ligand binding leads to a proteolytic cleavage that translocates the intracellular domain to the nucleus while the ECD is endocytosed with the ligand by the ligand-bearing cell [2,8]. Microscopically, CADASIL is characterized by the degeneration of VSMC and accumulation of granular osmiophilic material close to the VSMC [6]. The ligand Jagged-1 is primarily expressed on endothelial cells and VSMC and the loss endothelial Jagged-1 caused an impaired VSMC differentiation [9,10,11].

Brain magnetic resonance imaging is widely used for the differential diagnosis, assessment of the severity of illness, and predicting patients’ outcome in CADASIL [12,13,14]. However, it is costly for repetitive examination and is time-consuming for quantitative measurement, and uncooperative patients with advanced disease or patients with pacemakers or claustrophobia could not receive the examination. Therefore, blood biomarkers can have many valuable applications in CADASIL such as screening high-risk subjects for developing CADASIL, diagnosis of clinically probable disease without genetic evidence, prediction of clinical outcomes and prognosis, and surrogate endpoints in clinical trials [15,16]. Several promising blood biomarkers such as circulating progenitor cell and neurofilament light chain have been identified in CADASIL [17,18,19,20]. Unfortunately, they were not specific for CADASIL. Blood biomarkers related to the Notch signaling pathway have not been investigated extensively in CADASIL. It is noteworthy that the plasma level of NOTCH3 ECD (N3ECD) was lower significantly in transgenic mouse models of CADASIL than in the control mice [18]. Therefore, blood levels of N3ECD and Notch ligands could be useful for potential blood biomarkers for CADASIL. However, the normal physiological range of blood levels of N3ECD and Jagged-1 has not been determined yet. Therefore, for the purpose of their potential use as blood biomarkers, blood levels of N3ECD and the ligand Jagged-1 were measured in healthy individuals and the correlations with physiological and laboratory findings were investigated.

## 2. Results

### 2.1. Participant Characteristics

Of 279 participants, the mean age with standard deviation was 48.7 ± 10.6 and 47% were men. Current smoking and alcohol use was present in 24.7% and 73.8% of the participants, respectively. Table 1 summarizes the baseline characteristics of participants, including demographics, medical history, and laboratory values by gender.

### 2.2. Determination of Serum and Plasma Levels of N3ECD and Jagged-1

**Figure 1:**
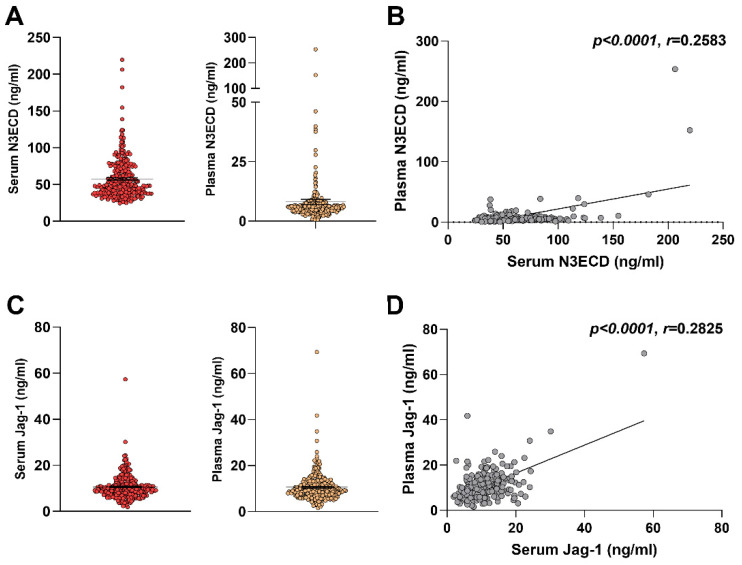
NOTCH3 extracellular domain (N3ECD) levels in healthy adults. N3ECD levels were measured in serum and plasma from healthy adults by ELISA (*n* = 279) (**A**) and a correlation between serum and plasma N3ECD levels was analyzed (**B**). Jagged-1 levels were measured in serum and plasma (**C**) and their correlation was assessed (**D**). Each symbol represents a value from a single subject; midline represents the mean; and error bars represent the standard error. The resulting Spearman’s correlation coefficient (*r*) and corresponding *p* value are reported.

**Figure 2:**
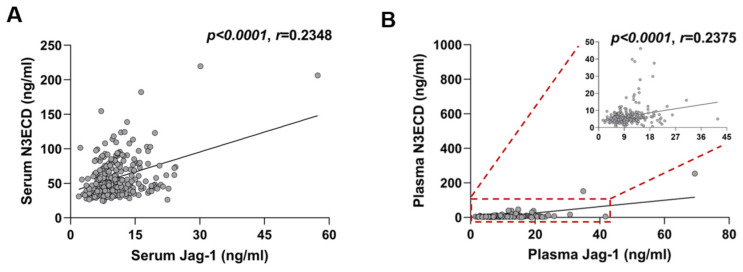
Correlations between N3ECD and Jag-1 levels. Positive correlations of serum (**A**) and plasma levels (**B**) between N3ECD and Jag1 (*n* = 279). Results are representative of three independent ELISA experiments. The resulting Spearman’s correlation coefficient (*r*) and corresponding *p*-values are reported.

Using ELISA technique, we measured the serum and plasma levels of N3ECD and Jagged-1 in healthy Korean people and the results are presented in Supplementary Table S1. The average coefficient of variation values of three independent experiments ranged from 3.03–5.60% for N3ECD and 1.25–4.87% for Jagged-1 (Supplemental Figure S1). The median serum N3ECD were significantly higher than plasma N3ECD level (median [interquartile range]; 49.42 [38.78–73.01] ng/mL vs. 5.73 [4.17–7.44] ng/mL, *p* < 0.001) (Figure 1A), and serum N3ECD level showed a positive correlation with plasma level (*p* < 0.0001, *r* = 0.2583) (Figure 1B). In contrast to N3ECD, the median serum and plasma levels of Jagged-1 did not differ significantly (9.74 [7.42–12.27] ng/mL vs. 9.39 [7.07–12.69], *p* = 0.998) (Figure 1C); however, they showed a weak positive correlation (*p* < 0.0001, *r* = 0.2825) (Figure 1D). The levels of N3ECD and Jagged-1 also showed weak correlations in both serum (*p* < 0.0001, *r* = 0.2348) and plasma (*p* < 0.0001, *r* = 0.2375) (Figure 2).

### 2.3. Associations between N3ECD or Jagged-1 Levels and Clinical Characteristics

**Figure 3:**
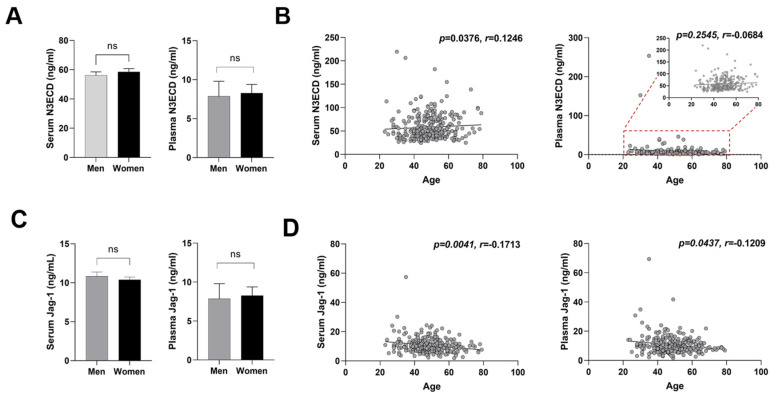
Comparison of mean N3ECD and Jagged-1 levels by gender and age. Serum and plasma N3ECD levels between male and female (**A**) and correlations between N3ECD levels and age (**B**). Jagged-1 serum and plasma levels in male and female (**C**) and correlations with age (**D**). Each symbol represents values from a single subject; midline represents the mean; and error bars represent the standard error. The resulting Spearman’s correlation coefficient (*r*) and corresponding *p*-value are reported. ns—not significant.

**Figure 4:**
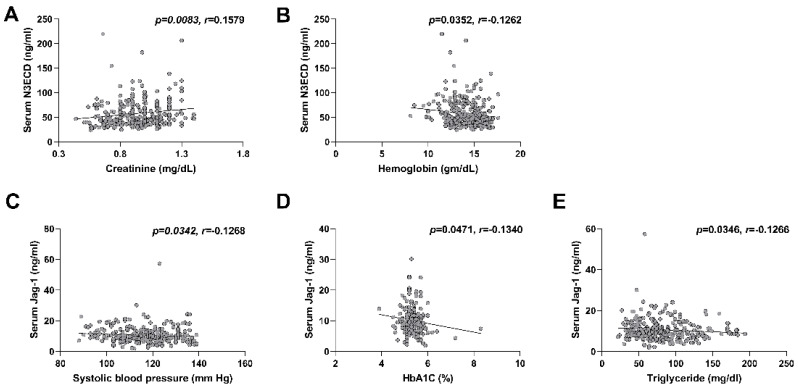
Correlations between serum N3ECD levels and laboratory values. Correlations of N3ECD with creatinine (**A**), and hemoglobin (**B**). Correlations of serum Jag-1 with systolic blood pressure (**C**), HbA1c (**D**), and triglyceride (**E**). Each symbol represents values from a single subject. The resulting Spearman’s correlation coefficient (*r*) and corresponding *p* value are reported.

We evaluated the association between serum and plasma levels of N3ECD or Jagged-1 and clinical characteristics, and the results are summarized in Table 2 and Table 3. There was no difference in serum and plasma N3ECD levels between men and women (Figure 3A). Serum N3ECD levels showed a very weak positive correlation with age (*p* < 0.0376, *r* = 0.1246), while plasma N3ECD levels did not show a correlation with age (*p* < 0.2545, *r* = −0.0684) (Figure 3B). Serum and plasma Jagged-1 levels also showed no difference between male and female (Figure 3C). In contrast to N3ECD, Jagged-1 levels showed very weak negative correlations with age in serum (*p* = 0.0041, *r* = −0.1713) and plasma (*p* = 0.0437, *r* = 0.1209) (Figure 3D). Among laboratory values, serum N3ECD levels only exhibited a weak negative correlation with hemoglobin (*p* = 0.035, *r* = −0.1262) and a negligible positive correlation with creatinine level (*p* = 0.0083, *r* =0.1579), while plasma N3ECD did not show correlations with any values (Figure 4A,B and Table 2). Serum Jagged-1 levels showed negative correlations with systolic blood pressure (*p* = 0.0342, *r* = −0.1268), HbA1c (*p* = 0.0471, *r* = −0.134) and triglyceride (*p* = 0.0346, *r* = −0.1266) (Figure 4C–E and Table 3), but again the size of correlation was very weak. After the adjustment for multiple testing, all the adjusted *p*-values were greater than 0.05 (Table 2 and Table 3).

## 3. Discussion

Several blood biomarkers have been suggested to monitor and predict the disease course in CADASIL patients. For example, the NfL blood level has been shown to correlate with the clinical burdens of patients with CADASIL [19] and can predict disease severity and long-term survival [18]. However, as a major component of the neuronal cytoskeleton in the axons, NfL is released into the blood upon any tissue damage in the central nervous system and therefore the level was also elevated in sporadic stroke and other various neurological disorders [21,22,23]. On the other hand, N3ECD has been known to accumulate in the VSMCs as a component of granular osmiophilic material in CADASIL patients even before the appearance of symptoms and signs of stroke [24,25], suggesting N3ECD as a promising biomarker that would be more sensitive in early stages. Therefore, as a preliminary study to investigate the potential of N3ECD and Jagged-1 as biomarkers for CADASIL patients, we measured N3ECD and Jagged-1 levels in healthy adult Koreans and tried to set up the reference range of them. We also evaluated changes in serum and plasma levels of these proteins according to various clinical characteristics, including age, gender, and blood chemistry and lipid profiles. As a result, we successfully established an ELISA assay and determined N3ECD and Jagged-1 levels in serum and plasma samples of healthy Korean adults.

Primo et al. have proposed three circulating proteins as biomarkers in a C445R mouse model of CADASIL: endostatin, high-temperature requirement A serine peptidase (HTRA1), and N3ECD [26]. They observed increased plasma levels of endostatin and HTRA1 in mice with the C455R mutation, whereas they saw decreased N3ECD level compared to controls. Interestingly, in three human samples they found that N3ECD levels were 3.6 times higher in the serum than in the plasma, which is consistent with our finding; the mean serum N3ECD level was seven times higher than the mean plasma level (Supplemental Table S1). Higher serum N3ECD levels may be attributed to cell lysis of white blood cells during the serum preparation, since Notch3 expression has been reported in human peripheral blood lymphocytes [27]. However, it is also possible that anticoagulant, ethylene–diamine–tetraacetic acid (EDTA), used for the routine preparation of plasma interferes with the detection of N3ECD, leading to lower levels in ELISA assay. Therefore, further study is required to determine which sample type is more appropriate for the measurement of N3ECD in the human blood.

We observed a tendency that serum N3ECD levels increase with age, although the degree of correlation was very weak. Considering that high Notch3 expression in adult human tissues seems to be restricted to vascular smooth muscle cells, it is likely that the aging process in small vessels affects expression of Notch3 and subsequently its serum levels. Although there are no studies examining Notch3 expression in vascular smooth muscle cells in aging vessels, decline in Notch signaling activity has been linked to impaired regenerative capacity of aged muscles, suggesting that altered Notch signaling occurs with aging [28,29]. Besides age, serum N3ECD correlated weakly with hemoglobin and creatinine levels in this study. Serum creatinine showed a positive correlation with age in this study. The concentration of serum creatinine rises progressively with age, especially after 60 years [30] and the decline in creatinine clearance accelerates with advancing age [31]. The decrease in creatinine clearance with age represents true renal aging. Therefore, the positive correlation between serum N3ECD and creatinine levels might be an aging effect. In contrast to N3ECD, Jagged-1 levels showed a weak negative correlation with age. These findings indicate that age needs to be corrected when analyzing N3ECD or Jagged-1 levels in human blood samples. Additionally, serum Jagged-1 showed correlations with systolic blood pressure, HbA1c, and triglyceride. However, as their size of correlation was mostly marginal, further studies with a larger number of samples including the patients who have abnormal laboratory values are needed to validate the results.

In summary, we successfully measured the levels of N3ECD and Jagged-1 using ELISA technique in a series of healthy human serum and plasma samples in this study. We documented that N3ECD levels are significantly higher in the serum than in plasma and tend to increase with age. In contrast, Jagged-1 levels had no difference between the serum and plasma levels and decreased with age. We believe our findings provide the basis for future research investigating the clinical use of N3ECD and Jagged-1 for CADASIL and other Notch signaling-related diseases.

## 4. Materials and Methods

### 4.1. Study Participants

Serum and plasma samples and their health information were provided by the Biobank of the Jeju National University Hospital, a member of the Korea Biobank Network. The informed consent was obtained from all participants at the time of sample donation to Biobank. Serum was prepared from the whole blood in a serum-separating tube (BD Vacutainer, Becton, Dickinson and Company, Franklin Lakes, NJ, USA) after the clot formation. The supernatant was collected after the centrifuging at 3400 rpm for 10 min at room temperature. Plasma was prepared from the whole blood in EDTA tube containing 5.4 mg of K2 EDTA (BD Vacutainer, Becton, Dickinson and Company, NJ, USA). The supernatant was collected after the cells have been removed by centrifugation at 4 °C. This study was approved by the Institutional Review Board of the Jeju National University Hospital (IRB File No. JEJUNUH 2020-08-010) and all procedures were in accordance with the Helsinki Declaration of 1964 and later versions.

The study participants consisted of 279 healthy Korean adults (131 men and 148 women; aged 23 to 79 years), who donated blood samples to the Biobank when they visited the hospital for routine health check-up. At the time of the check-up, the following information was collected from the participants: (1) height and weight; (2) past medical history (hypertension, diabetes mellitus, history of stroke, and history of ischemic heart disease); (3) systolic and diastolic blood pressure; and (4) laboratory values (white blood cell count, hemoglobin, platelet count, BUN, creatinine, fasting blood glucose, HbA1c, C-reactive protein, homocysteine, total cholesterol, LDL cholesterol, HDL cholesterol, and triglyceride). In this study, individuals with hypertension, diabetes mellitus, a history of stroke or ischemic heart disease were excluded.

### 4.2. Biochemical Analysis

N3ECD and Jagged-1 protein levels were determined in serum and plasma samples by sandwich enzyme-linked immunosorbent assay (ELISA). N3ECD ELISA is designed based on Primo, V. et al. [26]. Briefly, 96-well immuno-plates (SPL life science, South Korea) were coated with a monoclonal capture antibody (R&D Systems, Minneapolis, MN, USA; MAB 1559; 625 ng/mL in PBS) and were incubated overnight at 4 °C. Plates were blocked with 5% PBST (Tween 20; Sigma-Aldrich, St. Louis, MO, USA; P9416) at room temperature (RT) for 2 h. Recombinant Human Notch-3 Fc Chimera (R&D systems; 1559-NT) were used as a positive control standard protein. Recombinant protein was serially diluted from 100 ng/mL to 1.563 ng/mL and serum samples were diluted 1:10 in PBS. Standards and samples were loaded onto plates and incubated at RT for 2 h. Captured N3ECD was detected using biotinylated polyclonal antibody raised against the N3ECD (R&D Systems; BAF1559; 1000ng/mL). Detection antibody was diluted in Reagent Diluent Concentrate 2 (R&D Systems; DY995). After incubating plates at RT for 2 h, 100 µL of the working dilution of streptavidin-HRP (R&D Systems; DY992) was added to each well and incubated for 20 min at dark RT. The enzymatic reaction was developed with a 1-Step Ultra TMB: 3,3’,5,5’-tetramethylbenzidine-ELISA (Thermo Fisher Scientific, Waltham, Massachusetts USA; 34028) and then was stopped by adding 50 µL of stop solution (R&D systems; DY994) per well. The optical density was measured at 450 nm using a microplate reader (Tecan sunrise, Männedorf, Switzerland). We provided the range of N3ECD detection and the coefficient of variation for three independent ELISAs in Supplemental Figure S1. Ten-fold diluted serum and plasma N3ECD were founded to be in the range of 0~100 ng/mL. To measure Jagged-1 levels in serum, R&D systems Duoset (Cat# DY 1277) was used according to the manufacturer’s protocol. The detection range of Jagged1 was given as 0 to 40 ng/mL. In all analyses and figures, we used one of three independent ELISA experiments.

### 4.3. Statistical Analyses

Baseline demographic characteristics were compared between men and women using unpaired *t*-tests or χ^2^ tests according to the type of variables. For comparisons between serum and plasma levels of N3ECD and Jagged-1, a Wilcoxon signed-rank test was used. The correlation between N3ECD and Jagged-1 levels and different variables were tested using the Spearman’s rank correlation test. A *p*-value < 0.05 was considered statistically significant. For adjustment for multiple testing, we used the Holm-Šidák method. Statistical analyses were performed using GraphPad Prism software (version 9.0 GraphPad Software, Inc., San Diego, CA, USA; https://www.graphpad.com/scientific-software/prism, accessed on 7 January 2022).

## Figures and Tables

**Table 1:** Characteristics of 279 healthy subjects.

	Healthy Subjects (*n* = 279)		
	Male (*n* = 131)	Female (*n* = 148)	*p*-Value *
**Demographic characteristics**			
Age—yr(mean ± SD)	49.7 ± 11.0	47.8 ± 10.1	0.1319 *
Height—cm(mean ± SD)	171.2 ± 5.9	160.1 ± 5.4	<0.0001 *
Weight—kg(mean ± SD)	73.3 ± 8.7	57.1 ± 7.5	<0.0001 *
**Medical history—no.(%)**			
Smoking	60 (45.8)	9 (6.1)	<0.0001 ^†^
Drinking	111 (84.7)	95 (64.2)	<0.0001 ^†^
**Laboratory values—mean ± SD**			
Systolic blood pressure—mmHg	122.3 ± 9.6	112.7 ± 11.9	<0.0001 *
Diastolic blood pressure—mmHg	78.0 ± 6.9	71.6 ± 7.6	<0.0001 *
WBC	5.6 ± 1.2	5.1 ± 1.3	0.0027 *
Hemoglobin	15.3 ± 1.0	12.8 ± 1.2	<0.0001 *
Platelet	235.8 ± 47.1	258.3 ± 59.6	0.0007 *
BUN	13.1 ± 3.8	10.8 ± 3.5	<0.0001 *
Creatinine	1.1 ± 0.2	0.8 ± 0.1	<0.0001 *
FBS	92.7 ± 8.9 (*n* = 128)	88.2 ± 8.0 (*n* = 146)	<0.0001 *
HbA1c-%	5.5 ± 0.5 (*n* = 102)	5.3 ± 0.3 (*n* = 118)	0.0078 *
CRP	0.1 ± 0.3 (*n* = 123)	0.1 ± 0.1 (*n* = 44)	0.2013 *
Homocysteine-umol/L	9.9 ± 2.8 (*n* = 50)	7.1 ± 1.6 (*n* = 73)	<0.0001 *
Total cholesterol—mg/dL	193.6 ± 23.1	185.4 ± 25.9	0.0062 *
LDL cholesterol—mg/dL	124.7 ± 20.4 (*n* = 119)	105.4 ± 22.7 (*n* = 144)	<0.0001 *
HDL cholesterol—mg/dL	52.8 ± 9.9	66.3 ± 15.2	<0.0001 *

Data are mean ± SD. * unpaired *t*-test, † χ^2^ test. *p* value: male vs. female, Student *t*-test was used for continuous variables and χ^2^ test was used for categorical variables. Abbreviations: WBC = White Blood Cell; BUN= Blood Urea Nitrogen; FBS = Fasting Blood Sugar; HbA1c = Hemoglobin A1C, glycated hemoglobin; CRP = C-reactive protein; LDL = Low-Density Lipoprotein; HDL = High-Density Lipoprotein.

**Table 2:** Correlations between blood N3ECD levels and clinical characteristics.

N3ECD	Serum			Plasma		
	r *	*p* Value	*p* Value ^†^	r *	*p* Value	*p* Value ^†^
**Demographic characteristics**						
Age	0.1246	0.0376	0.4983	−0.0684	0.2545	0.9836
Sex		0.2927	0.9869		0.8529	0.9992
Height	−0.0588	0.3279	0.9874	−0.0341	0.5704	0.9992
Weight	−0.0790	0.1884	0.9644	−0.0256	0.6696	0.9992
**Medical history**						
Smoking		0.3366	0.9874		0.1253	0.9102
Drinking		0.9733	0.9989		0.5277	0.9992
**Laboratory values**						
Systolic blood pressure	−0.0809	0.1781	0.9644	−0.0080	0.8898	0.9992
Diastolic blood pressure	−0.0795	0.1853	0.9644	−0.0380	0.5175	0.9992
WBC	−0.0105	0.8619	0.9989	0.1056	0.0782	0.8038
Hemoglobin	−0.1262	0.0352	0.4938	−0.1076	0.2038	0.9672
Platelet	0.0228	0.7047	0.9989	0.0321	0.5925	0.9992
BUN	0.0337	0.5749	0.9989	0.0628	0.2958	0.9895
Creatinine	0.1579	0.0083	0.1535	0.0393	0.5130	0.9992
FBS	−0.0409	0.4998	0.9980	−0.0879	0.1467	0.9326
HbA1c (ngsp)	−0.0070	0.9180	0.9989	−0.0419	0.5361	0.9992
CRP	0.0330	0.5909	0.9989	−0.0521	0.3952	0.9976
Homocysteine	−0.0974	0.2837	0.9869	−0.0710	0.4319	0.9980
Total cholesterol	−0.0272	0.6506	0.9989	−0.0819	0.1752	0.9541
LDL cholesterol	−0.0788	0.2025	0.9644	−0.0981	0.1100	0.8908
HDL cholesterol	0.0218	0.7170	0.9989	0.0141	0.8061	0.9992

*r* *: Spearman correlation coefficient, *p* value ^†^: adjusted *p* value for multiple testing. Abbreviations: WBC = White Blood Cell; BUN= Blood Urea Nitrogen; FBS = Fasting Blood Sugar; HbA1c = Hemoglobin A1C, glycated hemoglobin; CRP = Creactive protein; LDL = Low-Density Lipoprotein; HDL = High-Density Lipoprotein.

**Table 3:** Correlations between blood Jag-1 levels and clinical characteristics.

Jag-1	Serum			Plasma		
	r *	*p* Value	*p* Value ^†^	r *	*p* Value	*p* Value ^†^
**Demographic characteristics**						
Age	−0.1713	0.0041	0.0789	−0.0129	0.0437	0.5722
Sex		0.9165	0.9914		0.3162	0.9895
Height	−0.1027	0.0867	0.7340	0.0223	0.5808	0.9996
Weight	−0.1035	0.0845	0.7340	0.0478	0.2362	0.9770
**Medical history**						
Smoking		0.0726	0.7223		0.8214	0.9999
Drinking		0.5385	0.9903		0.7700	0.9999
**Laboratory values**						
Systolic blood pressure	−0.1268	0.0342	0.4838	0.0138	0.7355	0.9999
Diastolic blood pressure	−0.1075	0.0730	0.7223	0.0248	0.5449	0.9996
WBC	−0.0868	0.1483	0.8655	0.0273	0.5033	0.9995
Hemoglobin	−0.0879	0.1430	0.8655	0.0479	0.2381	0.9770
Platelet	0.0878	0.1437	0.8655	0.0159	0.6939	0.9999
BUN	−0.0424	0.4808	0.9898	0.0011	0.9785	0.9999
Creatinine	0.0182	0.7619	0.9914	0.1020	0.0132	0.2334
FBS	−0.0141	0.8164	0.9914	−0.0108	0.7942	0.9999
HbA1c (ngsp)	−0.1340	0.0471	0.5804	−0.0916	0.0532	0.6262
CRP	−0.0712	0.2456	0.9403	0.0130	0.7601	0.9999
Homocysteine	−0.0357	0.6954	0.9914	−0.0071	0.9095	0.9999
Total cholesterol	−0.0358	0.5516	0.9903	0.0547	0.1765	0.9457
LDL cholesterol	−0.0551	0.3734	0.9762	0.0684	0.1012	0.8370
HDL cholesterol	0.0624	0.2992	0.9592	−0.0622	0.1266	0.8853

*r* *: Spearman correlation coefficient, *p* value ^†^: adjusted *p* value for multiple testing. Abbreviations: WBC = White Blood Cell; BUN= Blood Urea Nitrogen; FBS = Fasting Blood Sugar; HbA1c = Hemoglobin A1C, glycated hemoglobin; CRP = C-reactive protein; LDL = Low-Density Lipoprotein; HDL = High-Density Lipoprotein.

## Data Availability

Not applicable.

## Supplementary Materials

The following supporting information can be downloaded at: https://www.mdpi.com/article/10.3390/ijms231810547/s1.
