# Supplementary material for: Determination of Blood NOTCH3 Extracellular Domain and Jagged-1 Levels in Healthy Subjects
Source: Int J Mol Sci. 2022 Sep 11;23(18):10547. doi: 10.3390/ijms231810547 (PMC9505916; doi:10.3390/ijms231810547)
Supplement: Supplementary file 1 [file ijms-23-10547-s001.zip › ijms-1866287-supplementary.pdf]

Supplemental Table S1 . Blood N3ECD and Jag-1 levels of 279 healthy subjects

	N3ECD (ng/ml)		Jag-1 (ng/ml)	
	**Serum**	**Plasma**	**Serum**	**Plasma**
**Number of subjects (n)**	279	279	279	279
**25% Percentile**	38.78	4.166	7.419	7.074
**Median**	48.74	5.731	9.745	9.397
**75% Percentile**	71.42	7.435	12.27	12.69
**Mean**	57.61	8.08	10.62	10.62
**Std. Deviation**	27.51	18	5.312	6.193

Abbreviations: Std = standard

Supplemental Figure S1

A

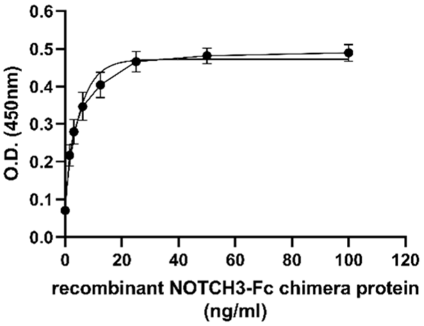

B

(n=279)	Average CV(%)			
	N3ECD		Jag-1	
	Serum	Plasma	Serum	Plasma
Experiment 1	3.92	5.35	3.43	3.67
Experiment 2	3.73	5.60	3.27	4.87
Experiment 3	3.03	4.21	3.6	1.25

Supplemental FigureS1.A. limit of N3ECD protein detection in ELISA method 4-PL of the N3ECD ELISA was found to range up to ~100ng/ml. B. Coefficient variation values of three independent ELISA experiments for N3ECD and Jag1 protein level in serum and Jag1. Four parameter logistic (4PL) curve is a regression model used to analyze ELISA.
